# Clinical presentation of intussusception in Swedish children under 3 years of age and the validity of diagnostic coding

**DOI:** 10.1007/s00383-018-4421-3

**Published:** 2018-11-26

**Authors:** Lina Schollin Ask, Jan F. Svensson, Ola Olén, Åke Örtqvist

**Affiliations:** 1 Sach´s Children and Youth Hospital, South General Hospital, Sjukhusbacken 10, 118 83 Stockholm, Sweden; 2 Department of Medicine, Clinical Epidemiology Unit, Karolinska Institutet, Stockholm, Sweden; 3 Department of Paediatric Surgery, Karolinska University Hospital, Stockholm, Sweden; 4 Department of Women’s and Children’s Health, Karolinska Institutet, Stockholm, Sweden; 5 Department of Communicable Disease Control and Prevention, Stockholm County Council, Stockholm, Sweden; 6 Division of Infectious Diseases, Department of Medicine, Solna, Karolinska Institutet, Stockholm, Sweden

**Keywords:** Children, Diagnostic coding, Intussusception, Rotavirus vaccine, Validation study

## Abstract

**Purpose:**

Intussusception has been associated with rotavirus vaccine. The rotavirus vaccine will soon be introduced in the Swedish national immunization program. A validation of the diagnosis of intussusception among Swedish children in the Swedish National Patient Register is needed, as a basis for future vaccine safety surveillance by Swedish registers.

**Methods:**

This diagnostic study reviewed the medical admission records of 392 Swedish children with intussusception from 1987 to 2013. The records were randomly selected by The National Board of Health and Welfare from all Sweden and from both pediatric and pediatric surgery care. Positive predictive values (PPV) were calculated to study the concordance between the diagnosis coded in the Swedish Patient Register and the accepted international criteria of case definitions.

**Results:**

The PPV for a definitive diagnosis, based on certain radiology findings or surgery, was 84%. When clinically probable cases were added the PPV was 87%. When cases of possible intussusception were added the PPV was 89%. The PPV for the 240 children under 1 year was 88%.

**Conclusion:**

Swedish health care registers can be used in the evaluation of incidences of intussusception when rotavirus vaccine will be introduced, due to a high validity of the diagnosis of intussusception in the registers.

## Introduction

Since 2013, the World Health Organization (WHO) has recommended all countries to vaccinate against the rotavirus infection, which is the most common global cause of severe diarrhea in children under 5 years of age [1]. By the end of 2016, the vaccination had been introduced into 90 countries [2].

In February 2017, the Swedish Government decided to include the rotavirus vaccination in the national child vaccination program. The national inclusion has not started yet but it has already been implemented in some regions, such as Stockholm County.

The rotavirus vaccination has been associated with an increased risk of intussusception [3–10] and the WHO has pointed out the importance of performing national surveys of this diagnosis when a country introduces the vaccine.

Intussusception is a severe condition, where the bowel folds into itself and causes an obstruction that could result in death if not treated [11]. In countries similar to Sweden it occurs most frequently in small children. The peak age is 4–8 months and the baseline incidence varies between 27 and 101 cases per 100,000 children who are less than 1 year old in high income settings [12]. It is more common in boys [11, 13] and the most frequent symptoms are described as the classic triad of vomiting, rectal bleeding or bloody stools, and intermittent abdominal pain, although a wide range of symptoms may appear [11]. Intussusception is treated by radiological techniques, using liquid contrast enemas or gas enemas, or by surgery [14, 15].

The diagnosis of intussusception has been validated in some countries, but not in Sweden [16, 17]. However, an international clinical case definition has been developed for the diagnosis of intussusception by the Brighton collaboration to standardize and facilitate the safety surveillance of the rotavirus vaccine [18, 19].

The primary aim of our study was to use these international criteria to validate the diagnosis of intussusception in the Swedish National Patient Register for children under 3 years of age. This would facilitate a future surveillance of the incidence of intussusception by the register.

## Materials and methods

### Study population

The National Board of Health and Welfare identified a random sample of 500 cases who were under 3 years of age and had received their first diagnostic listing of intussusception in the Swedish National Patient Register between 1987 and 2013. The records were randomly selected from all geographic areas in Sweden and from both pediatric and pediatric surgery departments, since there is a tradition in Sweden of seeking care for young children with abdominal pain in both types of clinics. Intussusception was defined according to the International Classification of Diseases, as 560A in the Ninth Revision and K56.1 in the Tenth Revision. We requested 500 medical records from the different hospitals around Sweden and received 392 (78%).

### The Brighton criteria for intussusception

**Fig. 1:**
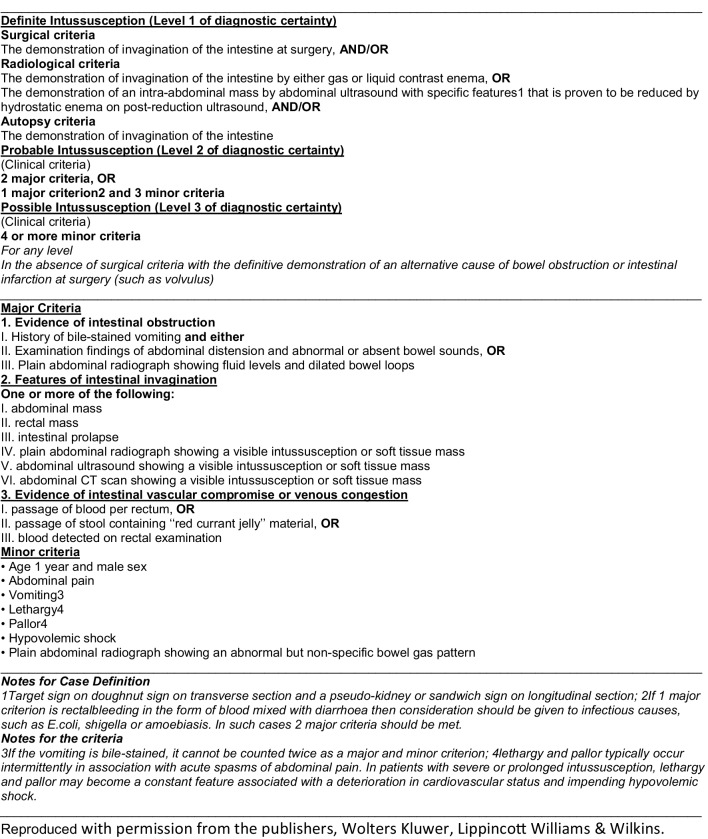
Clinical case definition for the diagnosis of acute intussusception by Bines et al

This study used the international clinical case definition of intussusception to categorize all medical records into the three different levels of diagnostic certainty: level one was definite, level two was probable and level three was possible intussusception [19] (Fig. 1). The criteria for this clinical case definition has a sensitivity of nearly 100% and specificity of 87–100% [19, 20].

### The Swedish National Patient Registry

Since 1987, the Swedish National Patient Register has achieved a complete coverage of all hospital admissions in Sweden and the validity has been shown to be high (85–95%) for several different diagnoses [21–23].

### Procedure

One of the authors, who is a pediatrician (LSA), reviewed the medical records according to the international criteria. When there was any doubt concerning any of the information, the medical records were discussed with another author (JFS), who is an experienced pediatric surgeon. They discussed 53 records and agreements were reached in each case.

### Statistical analysis

Based on earlier Swedish and European data [24, 25], we assumed that from 1987 to 2013 there would have been about 6000 cases of intussusception in Sweden in children who were less than 3 years of age. We estimated that records from 330 admissions would be enough to produce a generalizable estimated PPV of 80% with a 95% confidence interval (CI) with a precision of 4% either side of the PPV. From our earlier experiences in the research group, we knew that it was not possible to obtain all of the requested records and that we would probably lose about a third. That is why 500 records were requested.

Positive predictive values (PPVs) were calculated with 95% confidence intervals using binomial distribution [26] for each level of intussusception and any missing information on the variables was assumed to be negative in all the analyses. SPSS Statistics, version 22.0 (IBM Corp, Armonk, NY, USA), was used for the statistical analyses.

## Results

### Demographics of the study population

**Fig. 2:**
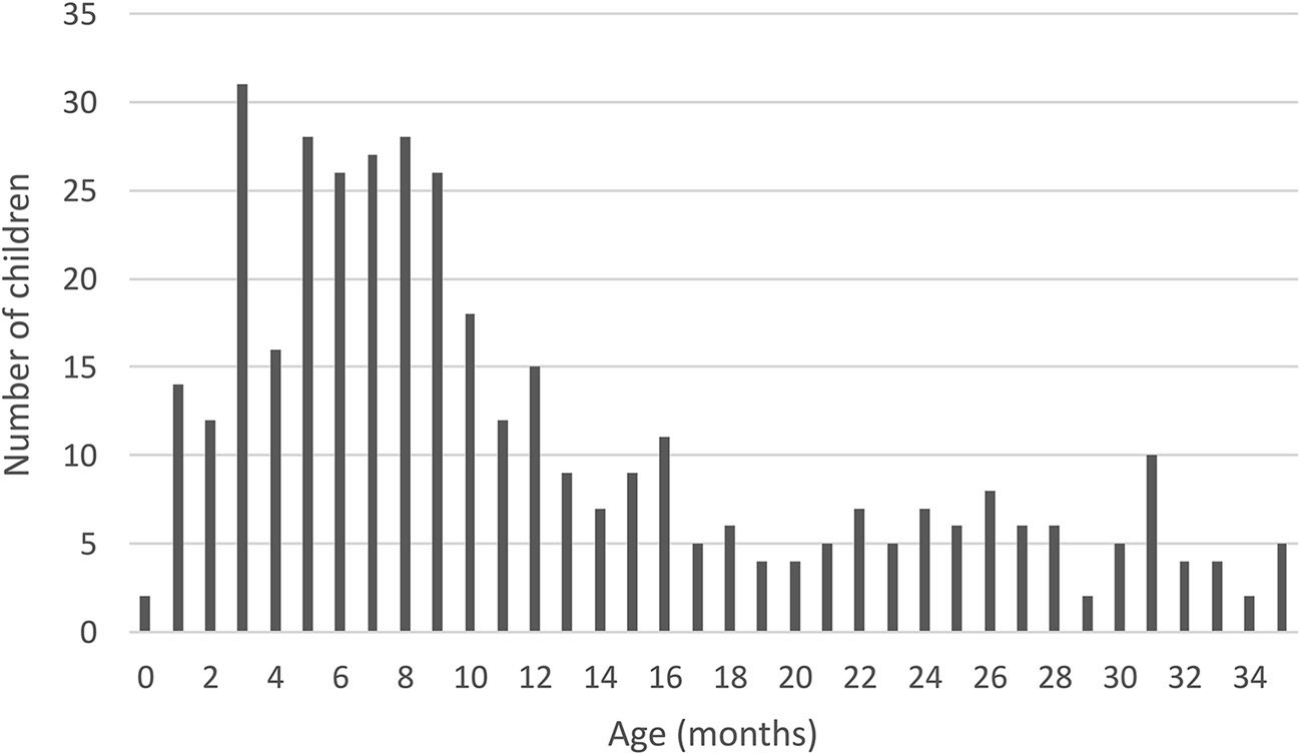
Age distribution for the study population of 392 children under 3 years of age with a diagnosis of invagination (ICD-9 560A or ICD-10 K56.1) in Sweden, 1986–2013

Table 1 presents the demographics of the study population. Medical records from the 392 admissions were reviewed and the majority were male, 262/392 (67%). With regard to age, 240/392 (61%) were less than 1 year old and 156/392 (40%) were between 3 and 9 months of age at the time of admission. The median age was 9 months (interquartile range 5–17 months). Figure 2 shows the age distribution of the study population. Admissions data showed that in 44/392 (11%) cases both a pediatric and a surgical department were involved, in 139/392 (35%) cases only a surgical department was involved and in 209/392 (53%) cases only a pediatric department was involved.

**Table 1:** Demographics of the 392 children with a diagnosis of intussusception

Variables	*N* (%)
Gender	
Boy	262 (67)
Girl	130 (33)
Age	
0–2 months	28 (7)
3–5 months	75 (19)
6–8 months	81 (21)
9–11 months	56 (14)
12–23 months	87 (22)
24–35 months	65 (17)
Time period	
1987–1996 (ICD-9 code 560A)	196 (50)
1997–2013 (ICD-10 code K56.1)	196 (50)
Type of department	
Surgical	139 (35)
Pediatric	209 (53)
Both	44 (11)
Total	392

### Positive predictive values

**Fig. 3:**
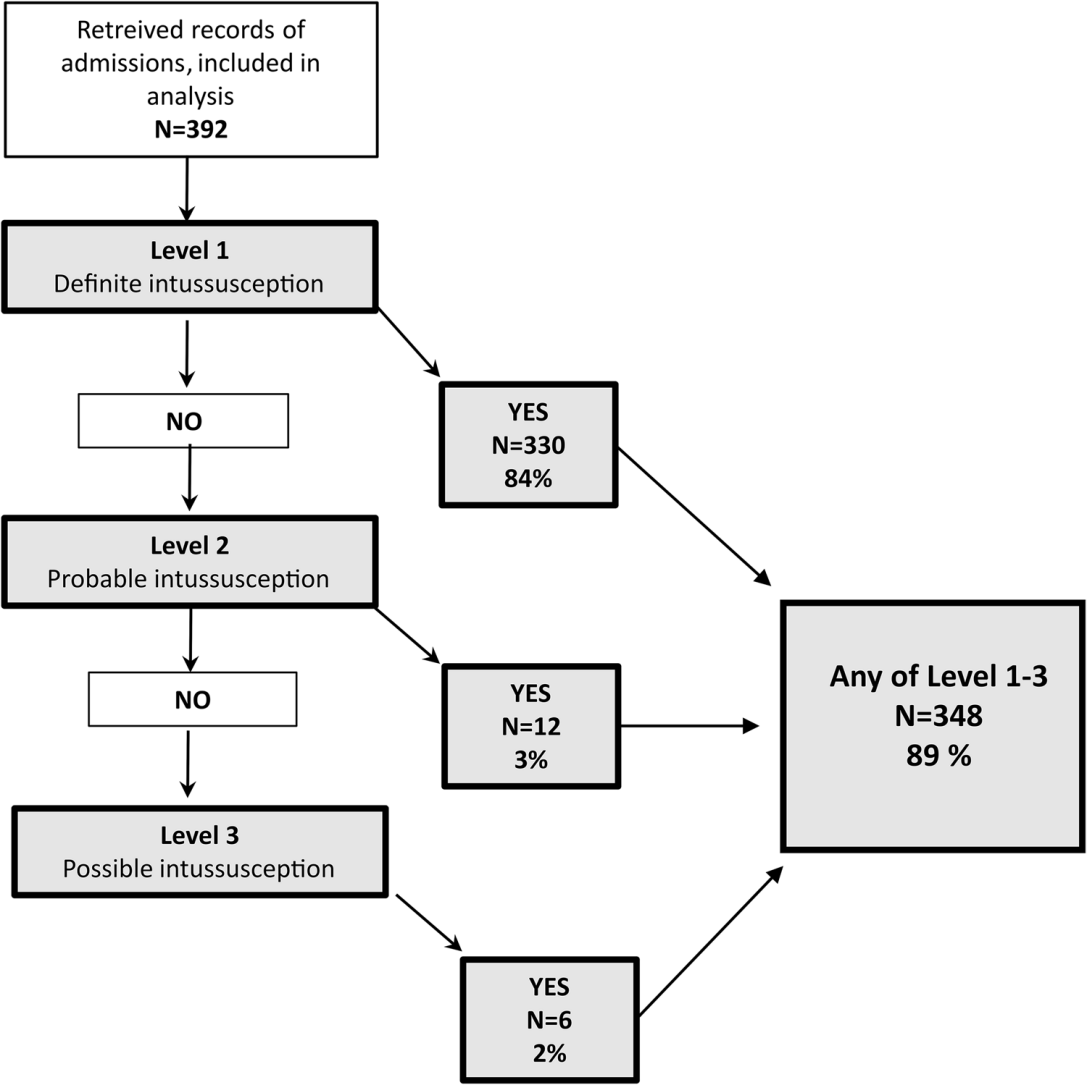
Procedure and main results

Figure 3 presents all different levels of PPV. In 348/392 cases, the Brighton collaboration case definitions of intussusception were fulfilled for at least one of the three levels of diagnostic certainty for intussusception (PPV 89%, 95% CI 86–92). When we reviewed the whole cohort, the criteria for definite intussusception (level one) were met by 330/392 children (PPV 84%, 95% CI 82–86) and by 12/392 (3%) for probable intussusception (level two) and 6/392 (2%) for possible intussusception (level three). When the PPV for levels one and two were combined, it was 87% (95% CI 84–90). In the subgroup of the 240 children who were less than 1 year old, the PPV for at least one of the three levels of diagnostic certainty for intussusception was 212/240 (88%) (95% CI 84–92).

### Clinical symptoms

All the symptoms and findings from radiology and surgery that appeared in the 392 cases are presented in Table 2. The most frequent symptom, in 346/392 (88%) of the cases, was a history or signs of abdominal pain and this was irrespective of the level of diagnostic certainty. Passing blood from the rectum was reported in 147/392 (38%) patients and some of those also had stools containing what was described as “red currant jelly” and/or blood detected during rectal examinations. Only 92/392 (23%) of the total cohort presented with all three symptoms of the classic triad, namely vomiting, abdominal pain and rectal bleeding or bloody stools.

**Table 2:** Symptoms, and radiology and surgical findings according to the Brighton Collaboration Case definitions in 392 admissions diagnosed with acute intussusception in Sweden during 1987–2013

Variables	Level 1 definite intussusception (*N* = 330) *N* (% of level 1)	Children < 1 year all levels (*N* = 240) *N* (% of all < 1 year)	Total all levels (*N* = 392) *N* (% of all)
Minor criteria			
Age < 1 year and male sex	128 (39)	155 (65)	159 (41)
Abdominal pain	296 (90)	204 (85)	346 (88)
Vomiting	236 (72)	176 (73)	257 (66)
Lethargy	179 (54)	126 (53)	194 (49)
Pallor	129 (39)	97 (40)	145 (37)
Hypovolemic shock	11 (3)	8 (1)	11 (3)
Plain abdominal radiograph showing an abnormal but non-specific bowel gas pattern	161 (49)	116 (48)	182 (46)
Major criteria			
History of bile-stained vomiting	23 (7)	22 (9)	23 (6)
Abdominal distension and abnormal or absent bowel sounds on examination	61 (18)	42 (18)	67 (17)
Plain abdominal radiograph showed fluid levels and dilated bowel loops	69 (21)	50 (21)	71 (18)
Abdominal mass	73 (22)	52 (22)	77 (20)
Rectal mass	2 (1)	1 (1)	2 (1)
Intestinal prolapse	2 (1)	2 (1)	2 (1)
Plain abdominal radiograph showed a visible intussusception or soft tissue mass	228 (69)	141 (59)	231 (59)
Abdominal ultrasound showed a visible intussusception or soft tissue mass	95 (29)	53 (22)	97 (25)
Abdominal CT scan showed a visible intussusception or soft tissue mass	2 (1)	1 (1)	3 (1)
Passing blood from rectum	127 (38)	120 (50)	147 (38)
Passing stools resembling ‘‘red currant jelly’’	30 (9)	32 (13)	37 (10)
Blood detected during rectal examination	29 (9)	27 (11)	32 (8)
Criteria related to level 1			
Surgical criteria*	84 (21)	62 (26)	84 (21)
Radiological criteria**	319 (97)	191 (80)	319 (81)
Autopsy criteria***	0	0	0

*CT* computerized tomography

*The demonstration of invagination of the intestine at surgery

**The demonstration of invagination of the intestine by either gas or liquid contrast enema, OR

The demonstration of an intra-abdominal mass by abdominal ultrasound with specific features1 that is proven to be reduced by hydrostatic enema on post-reduction ultrasound

***The demonstration of invagination of the intestine by autopsy

### Findings of radiology and surgery

Table 3 shows the results and findings of radiology and surgery in the study population. We found that 370 (94%) of the 392 children underwent a procedure with gas or liquid contrast enemas and 318/370 (86%) of these procedures were positive for a visible intussusception. It was possible to reduce the intussusception in 285/370 (77%) children when an enema was used. Ultrasound was performed on 107/392 (27%) children and 97/107 (91%) of these demonstrated visible intussusception or soft tissue mass. Ultrasound was performed less commonly during 1987–1999, (26/240, 11%) than during 2000–2013 (81/152, 53%) and in the last 2 years of the study, from 2012 to 2013, it was even higher (24/34, 71%). A visible soft tissue mass or intussusception was shown in 231/339 (68%) children who underwent a plain abdominal radiograph and 182/339 children (54%) had signs of abnormal but non-specific bowel gas patterns. Only 3/392 children (1%) had performed abdominal computed tomography and all of them had a visible intussusception.

**Table 3:** Number of cases where radiology was performed, based on the total study cohort of 392 children

Radiology performed, *n*/392 (%)	Abnormal but non-specific bowel gas pattern *N* (%)	Visible intussusception or soft tissue mass *N* (%)	Fluid levels and dilated bowel loops *N* (%)
Plain abdominal radiograph, 339 performed (86%)	182/339 (54)	231/339 (68)	71/339 (21)
Abdominal ultrasound 107, performed (27%)	–	97/107 (91)	–
Abdominal CT scan, 3 performed (1%)	–	3/3 (100)	–
Gas or liquid contrast enema, 370 performed (94%)	–	318/370 (86)	–

The radiological criteria for intussusception were fulfilled by 319/392 children (81%) children and in 318/319 cases it was carried out by gas or liquid contrast enema. In 73/318 (23%) of these children, it was not possible to reduce the intussusception and surgery was required. Ultrasound was used for the other case that fulfilled the criteria.

We found that 84/392 children (21%) fulfilled the surgical criteria. In 11 (13%) of these 84 cases, the child was in such a poor condition that surgery was performed without examining them with a gas or liquid contrast enema and 36/84 (43%) had signs of fluid levels and dilated bowel loops when a plain abdominal radiograph was performed. There were no deaths in the whole cohort of 392 children.

## Discussion

### Main findings

This nationwide Swedish study examined the validity of the coding for intussusception for children who were younger than 3 years old in the Swedish National Patient Register and found that it was high in 392 cases between 1987 and 2013. The PPV was 84% (95% CI 82–86) when typical radiological findings or surgery were required for a diagnosis of intussusception and up to 89% when clinically likely cases were also included. For the subgroup of children below 1 year of age the PPV was 88%.

### Comparisons with earlier studies

Our findings were in accordance with those from other countries presenting PPVs that fulfilled at least one of the three levels of diagnostic certainty for intussusception, which were 73% in Canada [17], 81–86% in Great Britain [27] and 86% in Switzerland [28]. Therefore, our results could be used as a platform for further studies of vaccine safety after the national introduction of the rotavirus vaccine in Sweden.

### Clinical symptoms

We demonstrated that children that were affected by intussusception presented with a wide range of symptoms, which emphasizes the importance of describing them as carefully as possible in medical records to facilitate later evaluation of the diagnoses. European studies have reported that the classic triad of symptoms, of vomiting, abdominal pain and rectal bleeding or bloody stools, were present in 10–66% of cases [11, 25]. In our study, the triad was present in 24% of the cases, while the most frequent symptom (88%) was abdominal pain, which was consistent with other studies [16, 20]. Abdominal pain has been shown to have a high PPV of 99%, a high negative predictive value of 100% and a high sensitivity 90%, but a specificity of just 19% when validated within the Brighton criteria for intussusception [20].

### Radiology

The Brighton collaboration criteria for intussusception, together with our results, emphasize the importance of using gas or liquid contrast enemas or abdominal ultrasound to verify the diagnosis, if the child is in a clinically stable condition. Most of the children in our study (94%) were examined by gas or liquid contrast enemas and 86% of these were positive for intussusception, compared to 65% who showed visible signs of intussusception when plain abdominal radiographs were performed.

Earlier Swedish studies showed that the success rate of enema therapy was more than 70% [29, 30], which was consistent with the 77% found in our study. Ultrasound examinations were only performed in 27% of cases, but of these cases 91% were positive for intussusception, indicating that this relatively harmless diagnostic method may have been underused. When we compared the oldest and most recent time periods during the 1987–2013 study, we found that a higher proportion of children underwent ultrasound during the later periods. The sensitivity of abdominal ultrasound in children affected by intussusception has been shown to be nearly 100%, while specificity has been reported to range from 78 to 100% [31, 32]. However, the performance of an abdominal ultrasonography depends on the operator and the quality of the performance can vary between hospitals and even during 1 day in the same hospital [15, 33].

### Surgery

In total, 21% of our children had findings of intussusception during surgery, which was similar to the 14% found in a European study [25] and the 28% in a study from the United States [12].

The treatment for intussusception is predominantly surgical in developing countries. The reason for this is that families often have to travel much greater distances to hospitals and children have a longer duration of symptoms and develop more severe conditions before a diagnosis of intussusception can be made [34].

### Strengths and limitations

One of the strengths of this study was the population-based design and the fact that it was based on a random sample from a nationwide patient register. Another strength was the use of standardized case definitions from the Brighton collaboration, which has been described as a reliable method when compared to non-systematic reviews [35–37].

However, there were some limitations to this study, including the retrospective study design, which meant that all of the available information depended on the quality of the reported information in the medical records. If information was missing on a variable in the case notes, it was analyzed as negative rather than being excluded. A previous study reported that using this procedure can dilute the results and lead to the PPV being lower than it should be [16]. Another limitation was that we depended on the reviewer´s interpretation of the symptoms and findings. In a previous study, different clinicians only agreed on the level of intussusception in 49% of cases, but there were no disagreements with regard to positive and negative diagnoses [20]. When the primary reviewing author in our study had any doubts about any information relating to a case, the medical record was discussed with a second reviewer until a consensus was reached. Furthermore, the radiology results were only available as written information, no radiological evaluation was performed and we did not have access to radiology procedure codings, which could have increased the PPV. However, in a similar validation study in Ontario in 2013 [17], PPV increased from 72.4 to 81.7% while sensitivity decreased from 89.3 to 71.2% when a procedure code for a diagnostic enema or enema reduction was added to the International Classification of Diseases codes. These were validated by the same case definitions used in our study. Finally, we assumed that all children with intussusception were taken care of in hospitals and a limitation was that it was difficult to capture children with spontaneously reduced intussusception [28]. In such cases, initial symptoms may resolve spontaneously after admission and all the results of the examinations will be negative [28]. Although such children should have been captured by the level two or three case definitions in our study, they might as well be validated as not being a case of intussusception [38]. A future challenge is to study the use of case definitions in cases of intussusception as an adverse effect of the rotavirus vaccine. Because the children affected by intussusception as an adverse event of rotavirus will be younger than the age when the incidence of intussusception naturally peaks, they will might show a different clinical picture because of their young age [38].

## Conclusions

The validity of the coding for intussusception in the Swedish National Patient Register among children who were younger than 3 years was high, at 89%. Our findings suggest that when the rotavirus vaccine is introduced to the national child vaccination program in Sweden, its safety can be monitored using the Swedish National Patient Register and coding the diagnosis as intussusception.
